# The C-terminal region of OVGP1 remodels the zona pellucida and modifies fertility parameters

**DOI:** 10.1038/srep32556

**Published:** 2016-09-07

**Authors:** B. Algarra, L. Han, C. Soriano-Úbeda, M. Avilés, P. Coy, L. Jovine, M. Jiménez-Movilla

**Affiliations:** 1 Department of Cell Biology and Histology, School of Medicine, University of Murcia, Campus Mare Nostrum and IMIB-Arrixaca, Murcia, Spain; 2 Department of Biosciences and Nutrition & Center for Innovative Medicine, Karolinska Institutet, Huddinge, Sweden; 3 Department of Physiology, Faculty of Veterinary, University of Murcia, Campus Mare Nostrum and IMIB-Arrixaca, Murcia, Spain

## Abstract

OVGP1 is the major non-serum glycoprotein in the oviduct fluid at the time of fertilization and early embryo development. Its activity differs among species. Here, we show that the C-terminal region of recombinant OVGP1 regulates its binding to the extracellular zona pellucida and affects its activity during fertilization. While porcine OVGP1 penetrates two-thirds of the thickness of the zona pellucida, shorter OVGP1 glycoproteins, including rabbit OVGP1, are restricted to the outer one-third of the zona matrix. Deletion of the C-terminal region reduces the ability of the glycoprotein to penetrate through the zona pellucida and prevents OVGP1 endocytosis. This affects the structure of the zona matrix and increases its resistance to protease digestion. However, only full-length porcine OVGP1 is able to increase the efficiency rate of *in vitro* fertilization. Thus, our findings document that the presence or absence of conserved regions in the C-terminus of OVGP1 modify its association with the zona pellucida that affects matrix structure and renders the zona matrix permissive to sperm penetration and OVGP1 endocytosis into the egg.

In species with internal fertilization, the oviduct is the site of gamete fusion and early cleavage of the zygote. Thus, gametes and early embryos are immersed in oviduct fluid (OF) which is a mixture of plasma exudate, epithelial cells secretion, and follicular fluid released during ovulation. Although oviducts have long been considered mere conduits for gametes and embryos, recent studies document their involvement in gamete maturation, sperm capacitation, sperm selection, prevention of polyspermy and embryo development^1^,^2^. Estrogen-dependent, oviduct-specific glycoprotein (OVGP1, also known as oviductin) is the major non-serum protein present in OF and its biological activity differs among species. It was reported to be necessary to overcome an early embryonic block in mice^3^; increase sperm capacitation and fertilization in cows^4^; increase sperm-egg binding^5^ and zona penetration rates in hamster^6^ and human^7^; enhance goat embryo cleavage and blastocyst formation, including a block to polyspermy^8^; and finally to increase the quality of matured and fertilized (IVM/IVF) cat embryo. In pigs, OVGP1 contributes to the control of polyspermy^9^, improves the efficiency of *in vitro* fertilization^10^ and increases the number of fertilized eggs that develop into blastocysts^11^. Taken together, these studies highlight the significant role of OVGP1 in reproduction, where it is necessary for normal fertilization and early embryo development. Consequently, OVGP1 would seem suitable for to be added into culture medium to improve assisted reproductive technologies. However, despite robust data documenting the contributions of OVGP1 to reproductive physiology, there are conflicting reports of its effects among species. For instance, pretreatment of oocytes with OVGP1 increased the number of sperm bound to the zona pellucida (ZP) in hamster^5^, but the opposite effect was observed in pig^9^. As yet, molecular mechanisms that would explain these differences and the role of OVGP1 in reproduction remain to be established.

OVGP1 belongs to the glycoside hydrolase 18 family of proteins, whose N-terminal chitinase catalytic domain is highly conserved based on hydrophobic cluster analysis and sequence comparisons. However, the N-terminal domain of OVGP1 lacks an essential glutamic acid residue and is thus enzymatically inactive^12^. The N-terminal region of mature OVGP1 shows a high degree of identity (77–84%) and similarity (86–90%) to that of other species. It contain a signal peptide, several post-translational modifications sites involved in secretion^12^ as well as a clathrin box associated with endocytosis^13^. Hamster OVGP1 has been reported to be endocytosed by developing blastocysts and has been hypothesized to undergo degradation through the ubiquitin-proteasome pathway^14^. In contrast, the C-terminal region has a low degree of identity (37–63%) and similarity (50–75%) as well as several insertions/deletions in its sequence. Interestingly, putative O-glycosylation sites are mainly located within this part of the protein, which contains mucin-type tandem-repeats. Hamster OVGP1 has six repeats of 15 aminoacids; human, baboon and rhesus monkey contain only 4, whereas and cattle, sheep and pig have incomplete or no tandem-repeat sequences. *In silico* analysis of deduced amino acid sequences identified a Class III PDZ-binding domain in human, baboon, porcine and bonnet OVGP1 glycoproteins, suggesting that OVGP1 is a component of a multi-protein complex^13^. Recently, comparison of mammalian OVGP1 amino acid sequences defined five distinct regions (A-E) that are differentially conserved among mammalian groups. Region A, which corresponds to the N-terminus, has a high degree of identity in monotremes, marsupials and placental mammals. Region B shows low identity among different mammals and contains multiple insertions/deletions. Region C is an insertion present only in the mouse, and region E is typical of human, chimpanzee and orangutan^2^ (Supplementary Material Fig. S1). All mammalian OVGP1 glycoproteins possess region A (archetypal of the N-terminal chitinase domain), but differ in the length of the core protein and the presence or absence of the other C-terminal regions. Despite *in silico* analyses, virtually nothing is known about the contribution of the different regions of OVGP1 to its biological activity, or whether the presence or absence of the C-terminus of OVGP1 affects its species-specific roles. For this reason, we examined, at both the molecular and physiological level the role of the C-terminal region of OVGP1 in fertilization.

## Results

### OVGP1 binds to ZP through the N-terminal region

**Figure 1:**
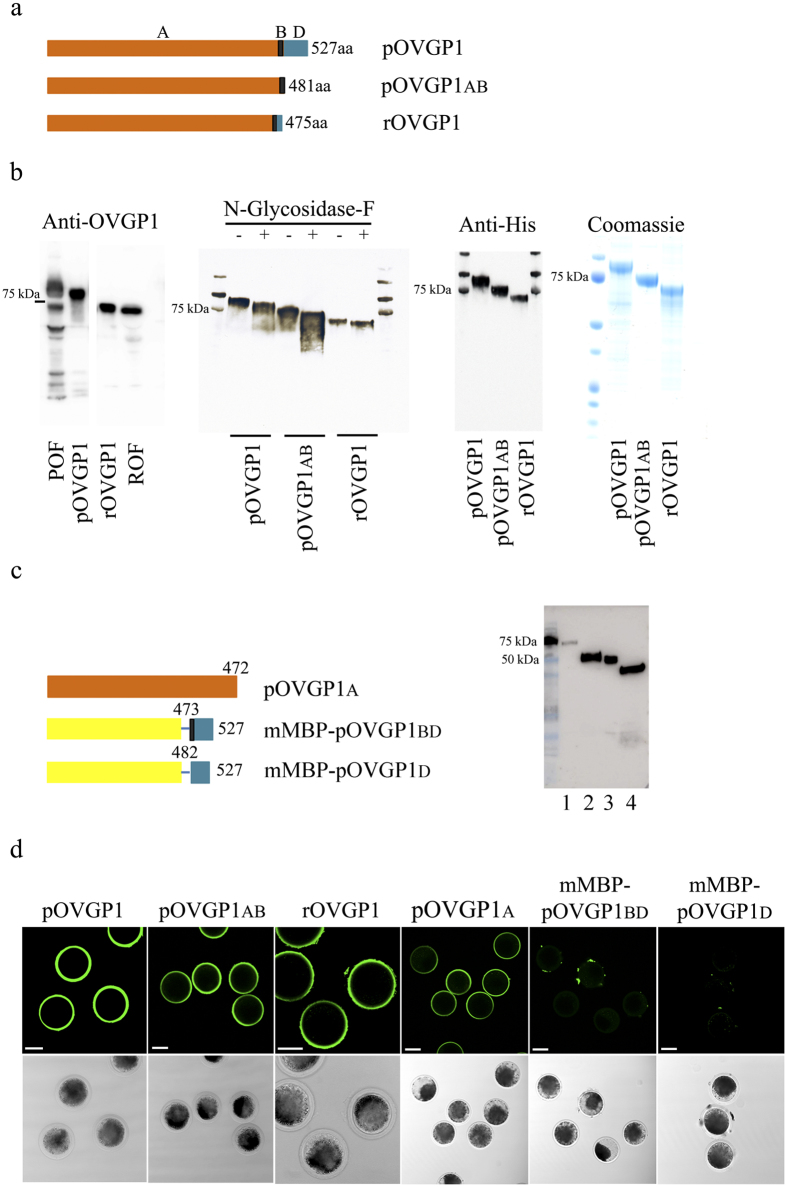
OVGP1 binds to the ZP through an N-terminal region. **(a)** Schematic representation of porcine (pOVGP1), porcine truncated (pOVGP1ab) and rabbit (rOVGP1) glycoproteins. A, B and D regions indicated. **(b)** Full-length porcine, rabbit and porcine truncated OVGP1 were expressed in HEK 293T cells. The proteins in conditional media were purified, separated on SDS-PAGE and either stained with Coomassie-Blue or transferred to a blotting membrane. Some proteins for western blotting were also treated with N-Glycosidase-F. The glycoproteins were detected with antibodies against OVGP1 or 6His tag. Porcine (POF) and rabbit (ROF) oviduct fluids were used as positive controls. **(c)** Schematic representation of porcine OVGP1 fragments corresponding to region A (pOVGP1a, lane 1), region BD (mMBP-pOVGP1bd, lane 2) and region D (mMBP-pOVGP1d, lane 3) which were inserted in-frame with mMBP (lane 4). Proteins were expressed in mammalian cells, separated by SDS-PAGE and analyzed by immunoblot using monoclonal antibody to the 6His tag. **(d)** IVM porcine oocytes were incubated for 60 min, at 37 °C with 125 μg/mL of pOVGP1, pOVGP1ab, rOVGP1 and with medium of transfected cells containing pOVGPa, mMBP-pOVGP1bd and mMBP-pOVGP1d. Oocytes were fixed and imaged by confocal fluorescence and DIC microscopy using monoclonal antibodies against the 6His tag. Scale bars, 75 μm.

To investigate the role of the OVGP1 C-terminal region, we expressed three histidine-tagged glycoproteins: full length OVGP1 of pig or rabbit and truncated pig OVGP1. Full-length pig OVGP1 (pOVGP1; Q28990) consists of 527 aa including regions A (1-472), B (473-481) and D (482-527)^2^ (Supplementary Material Fig. S1). Full-length rabbit OVGP1 (rOVGP1; Q95LB3) contains 475 aa where regions A (1-458) and B (459-467) are highly conserved with the pig, but region D is almost absent^2^. Finally, to analyze the biological role of C-terminal region D, a truncated pig OVGP1 variant lacking this region (481aa, pOVGP1ab) was also generated (Fig. 1a). The three proteins were expressed in Human Embryonic Kidney (HEK 293T) and Chinese Hamster Ovary (CHO) cells. Recombinant pOVGP1 had a slightly lower molecular mass (90 kDa) than native OVGP1 present in porcine OF (POF) (Fig. 1b, Supplementary Material Fig. S2) and its truncated pOVGP1ab variant had a molecular mass of 80 kDa on immunoblots probed with anti-OVGP1 and anti-His antibodies. Recombinant rOVGP1 had the same molecular mass (70 kDa) as native OVGP1 isolated from rabbit OF (Fig. 1b). Consistent with the observation that the three putative N-glycosylation sites of OVGP1 are located in region A (NetNGlyc 1.0 Server), its N-glycosylation did not depend on the presence of the D region, as shown by the fact that full-length pOVGP1 and pOVGP1ab displayed comparable molecular weight shifts upon digestion with N-Glycosidase-F (Fig. 1b). Thus, the 10 kDa molecular weight difference between the two proteins might be attributed to the absence of 50 amino acids and O-glycosylation sites within the D region. To perform physiological analyses, recombinant glycoproteins expressed in HEK 293T cells were purified by immobilized metal-affinity chromatography (IMAC) (Fig. 1b) and their identity was confirmed by mass spectrometry analysis (MS/MS) (Supplementary Material Fig. S3).

Recombinant polypeptides corresponding to each pOVGP1 region were also assayed for their ability to bind the ZP. The complete N-terminal region A open reading frame (ORF) was cloned, encoding 472 aa (pOVGP1a). Because of their small size, region BD (mMBP-pOVGP1bd) and region D (mMBP-pOVGP1d) were expressed as C-terminal fusions to a recently described maltose-binding protein variant that is efficiently secreted by mammalian cells (mMBP)^15^. N-terminally 6His-tagged recombinant pOVGP1a (70 kDa), mMBP-pOVGP1bd (50 kDa) and mMBP-pOVGP1d (48 kDa) proteins were detected in the conditioned medium of transfected cell medium (Fig. 1c). Also in this case, mass spectrometry was used to verify the identity of the recombinant polypeptides (Supplementary Material Fig. S3).

Full-length recombinant glycoproteins from pig and rabbit bound to the ZP of IVM porcine oocytes as did C-terminally deleted recombinant glycoproteins pOVGP1ab and pOVGP1a. However, mMBP fusion proteins containing the BD or D domains were not detected in the ZP (Fig. 1d, Supplementary Material Fig. S3). These results indicate that the A region is required for binding of OVGP1 to the ZP.

### The C-terminal region of OVGP1 modulates its binding to the ZP

**Figure 2:**
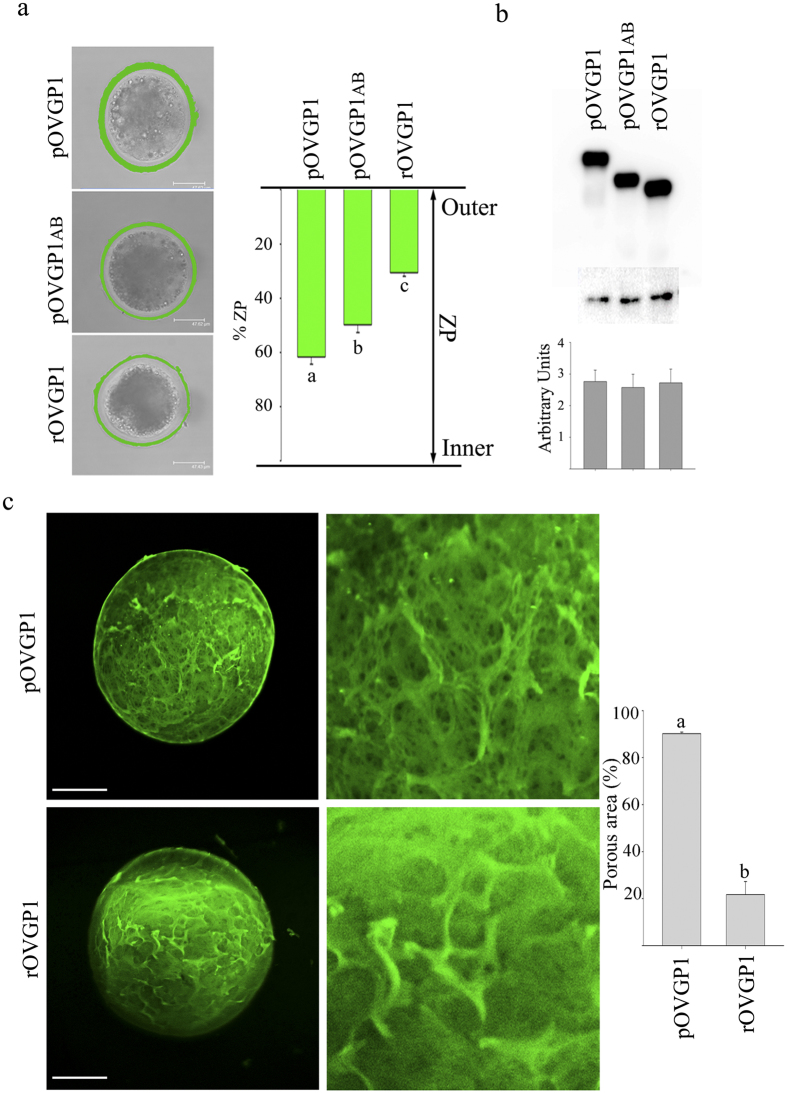
The C-terminus of OVGP1 modulates remodeling of the ZP. (**a**) Confocal immunofluorescence of IVM porcine oocytes. Representative photographs of mid-plane optical sections of oocytes incubated *in vitro* with 125 μg/mL of pOVGP1, pOVGP1ab and rOVGP1 (left). Graph (right) reflects the mean ± SEM (15 oocytes) of the zona penetration of the indicated protein. The letters (**a,b,c**) indicate significant differences, P < 0.01. **(b)** IVM porcine oocytes (20) were incubated for 1 hr at 37 °C in 125 μg/mL of pOVGP1, pOVGP1ab and rOVGP1, lysed and analyzed by immunoblot with anti-OVGP1 antibody. Anti-β actin was used as a loading control (top). Graph (bottom) reflects the mean ± SEM of recombinant protein in arbitrary units from three experiments. **(c)** Confocal immunofluorescent images of the zona surface after incubation with pOVGP1 and rOVGP1 (left). The graph (right) represents the mean ± SEM for the percentage of porous area in 8 images, p < 0.001.

Immunofluorescence analysis of IVM oocytes incubated with equal amounts of purified recombinant glycoproteins showed that full-length pOVGP1 penetrated 62% of the width of the zona matrix, whereas truncated glycoprotein pOVGP1ab penetrated only 50% (Fig. 2a). In the case of rabbit OVGP1 (rOVGP1) where domain D is almost absent, 30% penetration of the zona width was observed (Fig. 2a). Based on these observations, we expected that the ZP would retain a larger amount of glycoprotein after incubation with pOVGP1. However, equivalent amounts of protein were recovered from oocytes incubated with each of the three glycoproteins (Fig. 2b). This suggests that, although OVGP1-ZP penetration differs among proteins, comparable amounts of protein are retained within the zona matrix. Therefore, oocytes incubated with OVGP1 from rabbit should possess a denser layer of glycoprotein in the outer aspects of their zonae. This hypothesis was confirmed by imaging the surface of the ZP by confocal microscopy (Fig. 2c) which showed a mesh-like structure with numerous fenestrations formed by pores and hollows when oocytes were incubated with pOVGP1. These observations indicate that the glycoprotein permeated the 3D scaffold of the matrix. The ZP of oocytes incubated with rOVGP1 showed a spongy surface with few or no porous areas. Most of the surface pores appeared to coalesce to form a smooth surface. Glycoproteins seemed to be stacked on the surface of the matrix forming a protein layer to block pores. The porous surface observed in oocytes incubated with pOVGP1 was significantly higher than oocytes incubated with rOVGP1 (Fig. 2c, Supplementary Material Fig. S4). Thus, we suggest that the absence of OVGP1 C-terminal region D affects the matrix structure observed by increased ZP resistance to the protease digestion (see below).

### Deletion of region D prevents OVGP1 endocytosis by the oocyte

**Figure 3:**
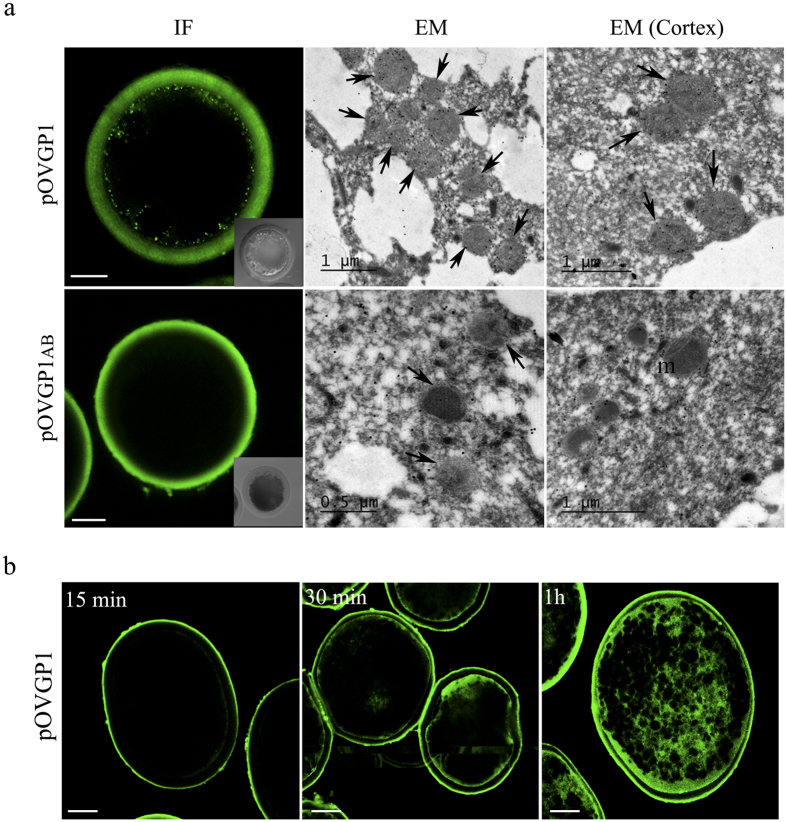
Recombinant pOVGP1 is endocytosed by IVM porcine oocytes. (**a**) IVM porcine oocytes were incubated with pOVGP1 and pOVGP1ab, fixed and permeabilized. Immunofluorescence (IF) confocal microscopy with anti-His antibody (left) was used to detect endocytosis in the cortex. Insets are DIC images. Scale bars, 30 μm. Immunogold transmission electron microscopy (EM) using anti-OVGP1 antibody, pOVGP1 was used to detect recombinant protein within multivesicular-like bodies (arrows) inside oocytes (middle) and in the cortex (right). Scale bars: 0.5 and 1 μm. m = mitochondria. **(b)** IVM porcine oocytes (15) were incubated for 15 min, 30 min or 1 hr in medium containing pOVGP1, fixed and permeabilized. Confocal inmunofluorescence with anti-His antibody was used to observe endocytosis. Scale bars, 20 μm.

Previous immunolocalization studies showed an association of OVGP1 with microvilli and membranes of unfertilized oocytes and blastomeres within the oviduct of different species^16^,^17^,^18^. Moreover, the presence of OVGP1 in multivesicular bodies of preimplantation embryos^14^ suggests OVGP1 cell incorporation by endocytosis. Here, permeabilized IVM oocytes incubated with pOVGP1 for 1 hr showed a specific dotted labeling in the cortex of the ooplasm (Fig. 3a). Moreover, by immune-gold transmission electron microscopy, pOVGP1 was detected within multivesicular-like bodies located inside the oocyte and in the cortex (Fig. 3a and Supplementary Material Fig. S5). Consistent with these observations supporting endocytosis, pOVGP1 incubation showed progressive glycoprotein accumulation in the oocyte cortex over time (Fig. 3b). However, no signal was detected inside oocytes incubated with pOVGP1ab lacks domain D (Fig. 3a). In addition, no immunostaining was observed when A, BD and D regions were incubated with the oocyte (Supplementary Material Fig. S6), suggesting that the entire protein must be present for complete penetration through the zona matrix and endocytosis by the oocyte.

### Effect of recombinant OVGP1 glycoproteins on ZP resistance to proteolysis

**Figure 4:**
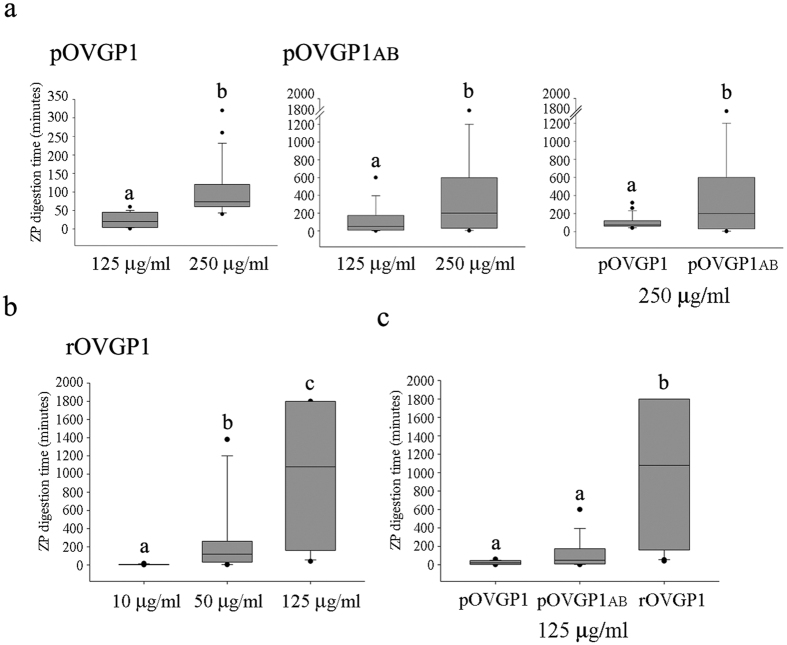
Effect of recombinant OVGP1 on ZP resistance to protease digestion. Box plots of digestion time of IVM porcine oocytes incubated in **(a)** 125 and 250 μg/mL (pOVGP1, pOVGP1ab) and **(b)** 10, 50 and 125 μg/mL (rOVGP1) for 60 min and transferred to a pronase solution (0.5% wt/vol in PBS). **(c)** Same as **(a)**, but with each OVGP1 protein at the same concentration (125 μg/mL). Boxes include the middle two quartiles with the median (horizontal line) and data points within the 10^th^ and 90^th^ percentiles (error bars). Outliers are indicated by dots. Experiments were carried out in triplicate and each group consisted of 20–30 oocytes. The letters (a, b, c) in each graph indicate significant differences, p < 0.05.

OVGP1 has been referred to as one of the OF components involved in enzymatic ZP hardening, whereby ZP acquires resistance to proteolytic digestion during transit in the oviduct^9^. To determine the influence of OVGP1 on ZP hardening, IVM oocytes were exposed to 125 and 250 μg/ml of pOVGP1 and pOVGP1ab for 1 hr before testing the resistance of the ZP to proteolytic digestion. The ZP of porcine oocytes exposed to pOVGP1 at 125 and 250 μg/ml were dissolved by pronase in 24 (24.1 ± 3.3) and 102 (102 ± 11.4) min, respectively (Fig. 4a), suggesting a dose-dependent effect of OVGP1 on ZP resistance to enzymatic proteolysis. In the case of oocytes exposed to porcine pOVGP1ab, the resistance of ZP to proteolysis increased to 112 (125 μg/ml, 112 ± 25.6) and 464 (250 μg/ml, 464.9 ± 73.5) minutes, respectively (Fig. 4a). Rabbit OF had the greatest effect on ZP resistance to proteolysis among the species tested^19^. The ZP of IVM porcine oocytes exposed to rOVGP1 showed digestion times of 6.4 ± 0.8 min (5 μg/ml), 312 ± 63.1 min (50 μg/ml) and 989 ± 151.4 min (125 μg/ml) (Fig. 4b). At the same protein concentration, rOVGP1 made the ZP more resistant to pronase digestion than pOVGP1 or pOVGP1ab (Fig. 4c). In the case of oocytes exposed to rOVGP1, some ZPs exhibited 2 days of resistance to pronase digestion.

### Only pig OVGP1 increases fertilization efficiency

**Figure 5:**
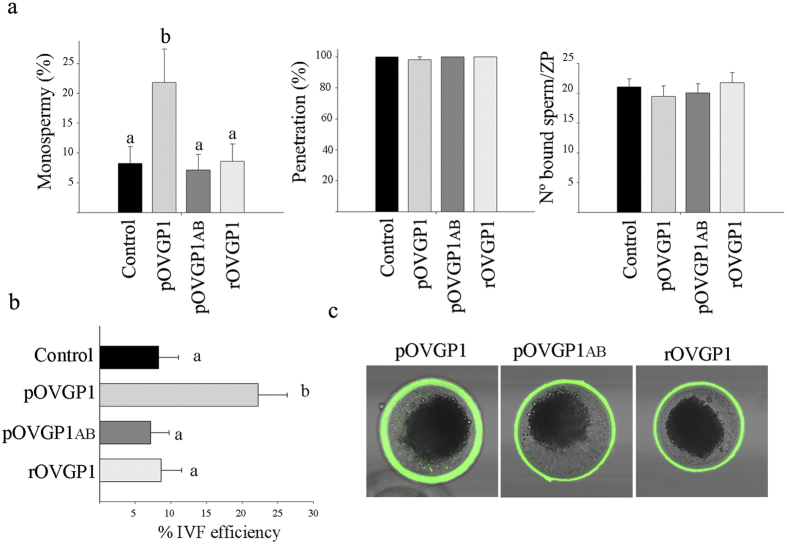
Effect of incubation with pOVGP1, pOVGP1ab and rOVGP1 on IVF results. (**a**) In porcine IVF, monospermy (left), penetration (middle) and number of bound sperms per ZP (right) were evaluated. Experiments were carried out in triplicate with 25–30 oocytes per group. Mean ± SEM. **(b)** IVF efficiency (%) determined by the percentage of penetrated oocytes that were monospermic. **(c)** Confocal immunofluorescence of IVM porcine oocytes after IVF to detect protein bound to the ZP. Note the dotted label only in cytoplasm of the oocytes treated with pOVGP1. The letters (**a,b**) in each graph indicate statistically significant differences, p < 0.01.

IVF rates of eggs exposed to different recombinant glycoproteins before and during insemination were measured using IVM porcine eggs. Full-length pOVGP1glycoprotein significantly increased the percentage of monospermic fertilization (21.8 ± 5.6) compared with rOVGP1 (8.6 ± 2.9) or pOVGP1ab (7.1 ± 2.6). The percentage of monospermic zygotes exposed to rOVGP1 and pOVGP1ab was similar to that of the control group (8.3 ± 2.8) (Fig. 5a). However, no significant differences were observed in the number of spermatozoa bound to the ZP and percentage of penetration between eggs exposed to the different recombinant glycoproteins and control (Fig. 5a). Those results suggest that only full-length, pOVGP1 is able to increase the efficiency of IVF as measured by monospermic fertilization which increased from 8% (control) to 23% (Fig. 5b). It is important to note that the recombinant glycoproteins were retained within the ZP when oocytes were used to assess IVF parameters (Fig. 5c). Taken together, these data show that recombinant porcine OVGP1 can be easily purified in a biologically active form that can be added to IVF media to enhance the rate of fertilization.

## Discussion

Of glycoprotein OVGP1 is present at the moment of fertilization and during the early stages of embryonic development in a wide range of mammals^12^. The effects of OVGP1 on reproduction are mediated primarily through interactions with the ZP, the specialized extracellular matrix of the oocyte that controls sperm binding and penetration. The detection of OVGP1 in multivesicular bodies of preimplantation embryos^14^ indicates that the protein is subsequently endocytosed. However, a challenge in interpreting the results of these studies are variations in the interaction between OVGP1 and the ZP, which leads to physiological discrepancies among species. Rapid adaptive evolution of the OVGP1 gene among mammals^20^ has been reported to promote the divergence of the oviduct protein. Analyses of the amino acid sequence of OVGP1 showed that its N-terminal region is much more conserved than its C-terminus, leading to the hypothesis that the latter constitutes both a binding and species-specific recognition domain. In this study, we investigated the effect of different OVGP1 C-termini on fertility. Our results reveal that the C-terminal sequence of OVGP1 influences the capacity of the protein to penetrate the ZP and its presence is required for OVGP1 to be endocytosed by the oocyte. This has an effect on the structure of the ZP, which is reflected by changes in its sensitivity to pronase digestion and fertilization success.

A comparison of the amino acid sequences of various mammalian OVGP1 showed that the pig protein contains three conserved regions (A, B and D)^2^ (Supplementary Material Fig. S1). Moreover, rabbit OVGP1 is 52 amino acids shorter than porcine OVGP1 as a result of the almost complete loss of region D. To analyze the biological function of region D at the C-terminus, a truncated porcine OVGP1 recombinant glycoprotein was generated. All the recombinant OVGP1 glycoproteins that contained N-terminal region A maintained their capacity to bind to the ZP. These data suggest that region A contains a ZP-binding domain, perhaps based on its chitinase-like domain that may retain sugar-binding properties to associate with gametes^21^. In addition, such region could be involved in molecular interactions with other oviductal proteins^13^,^22^, generating the microenvironment necessary to facilitate fertilization and early embryo development.

The ZP of ovarian oocytes undergoes maturation during folliculogenesis in preparation for encountering spermatozoa in the female reproductive tract^23^,^24^; moreover, several studies have reported further modifications during the transit through the oviduct^9^,^25^,^26^,^27^. Some investigators^28^,^29^,^30^ describe a net-like porous surface in mature oocytes with a rough pattern on the surface of the zona matrix while immature oocytes have a compact zona surface without pores^31^. These modifications occur while ovulated oocytes are immersed in OF, but the underlying molecular mechanisms are unknown. Here, we showed that porcine OVGP1 penetrates more than half of the width of the ZP and observed persistence of pore-like structures, indicating that the recombinant glycoprotein had penetrated the zona matrix in agreement with the morphology described for mature oocytes. However, lack of region D impaired the penetration ability of pOVGP1, so that the recombinant protein was limited to the surface of the matrix whereas rOVGP1 occupied most of the ZP pores and appeared stacked on the surface of the matrix. These results suggest that OVGP1 is involved in oviduct maturation of the ZP by affecting the morphology of the matrix and modulating OVGP1-ZP binding via region D.

The discrepancy observed between the ZP penetration abilities of porcine and rabbit OVGP1s may have physiological significance. The absence of endocytosis of truncated OVGP1 is due to its retention in the outer region of the ZP and the presence of the OVGP1 in multivesicular-like bodies in IVM oocytes (this manuscript) and in blastomeres of developing embryos^14^ could help explain the influence of OVGP1 on early stages of development^3^,^8^,^9^. Moreover, chitinase-like proteins such as HCgp-39 have been reported to initiate a signaling cascade in the connective-tissue, which leads to increased cell proliferation^32^,^33^. Similarly, OVGP1 could influences embryonic cell or blastomere development, with its C-terminus needed for order-specific effects among mammals. Notably, antibodies against a C-terminal peptide of OVGP1 inhibit early mouse development, so that and embryos do not progress pass the 2-cell stage^3^.

OVGP1 is considered to be one of the OF components involved in the enzymatic hardening of the ZP, which ZP acquires resistance to proteolytic digestion during oocyte transit in the oviductal tube^9^. The present study demonstrates that OVGP1 counteracts ZP hardening, a process that is modulated by its C-terminus. These observations are consistent with recent data reporting that rabbit OF produces the largest increases in ZP resistance among mouse, rat, hamster, rabbit, sheep, goat, pig and cow^34^. We have shown that oocyte incubation with shorter OVGP1 proteins increases the digestion time needed to degrade the ZP. Thus, under physiological conditions, region E in human or region C in mice could promote ZP penetration and decrease ZP hardening in humans or mouse ZP^35^.

The ability of the OF to induce ZP hardening is recapitulated with recombinant porcine OVGP1 and is proportional to its ability to facilitate monospermy in pig IVF^34^. Although we anticipated that truncated porcine OVGP1 and rabbit OVGP1 would increase the efficiency of fertilization, no effect was observed in pigs. Thus, the correlation between the induction of hardening and improved IVF rates only occurs when region D is present. Therefore, we attribute an order-specific role in modulating sperm binding, penetration and fertilization to the C-terminus of OVGP1. In support of this conclusion, human OVGP1 enhances sperm binding to the ZP, whereas heterologous OVGP1 (baboon) inhibits that effect even though the two proteins are 94% identical^7^,^36^; this may reflect the additional E domain at the human C-terminus. In contrast, when heterologous OVGP1 proteins (porcine and bovine) share the same conservative regions (A, B and D) (Supplementary Material Fig. S1), they had the same positive effect on IVF^9^. Taken together, these data suggest that the presence or absence of specific regions in the C-terminus of OVGP1 affects its association with the ZP as well as its ability to remodel of the matrix.

Structural changes in the zona matrix have been observed during egg development and post fertilization in a variety of animals^37^,^38^. However, in the absence of high-resolution structural information on ZP filaments, we lack a complete understanding of the structural basis of gamete interaction. In *Xenopus laevis*, the fertilization competence of the egg-coat envelope is regulated by direct interaction between dicalcin and gp41, a frog orthologue of mammalian ZP component ZP3^39^. Dicalcin might act as a key regulatory protein involved in mediating fertilization competence by modifying the three dimensional structure of egg coat ZP filaments^40^. Likewise, OVGP1 might generate a structural microenvironment necessary for sperm to reach the egg plasma membrane.

In conclusion, we propose a model in which OVGP1 binds to ZP via its highly conserved A region, which may also provide an anchor for additional oviduct proteins. The C-terminal regions of OVGP1 modulate its binding to the ZP, regulate OVGP1 activity and account for the reproductive role of OVGP1 in different mammalian orders. In pig, OVGP1 remodels the ZP structure by affecting zona hardening, as well as the ability of sperm to penetrate the zona matrix. More importantly, the presence of the D region at the C-terminus ensures OVGP1 endocytosis during the passage through the oviduct, before and after fertilization. Recombinant porcine OVGP1 improves fertilization efficiency in pigs abetted by its C-terminal region. By extending these findings to other species including humans, it may be possible to modulate mammalian fertilization by manipulating the presence/absence of regions D, C or E in the C-terminus of OVGP1. This open the exciting possibility of functionally exploring the correlation between C-terminal regions and order-specific effects on mammalian reproduction.

## Methods

### Construction of expression plasmids

mRNA was extracted from frozen porcine and rabbit oviducts with RNAqueous^®^ phenol free total RNA Isolation kit (Ambion^®^, Huntigton, United Kingdom). Reverse transcription produced single-stranded cDNA using the Superscript^®^ First Strand Synthesis System for RT-PCR (Invitrogen, Carlsbad, CA) according to the manufacturer’s instructions.

To obtain the full-length ORF of pOVGP1 (nucleotides 11–1594), including the 21-residue signal sequence, cDNA was PCR-amplified using two primers (pOVGP1F, pOVGP1R), each containing a*Kpn*I or a*Mun*I site. For generating C-terminally truncated constructs pOVGP1ab (corresponding to nucleotides 752–1453) and pOVGP1a (nucleotides 752–1426), two sets of primers were used (pOVGP1abf, pOVGP1abr, pOVGP1af, pOVGP1ar) that contained a *Xho*I site. cDNA from rabbit oviduct was used to PCR amplify the full-length rabbit OVGP1 ORF (rOVGP1; nucleotides 12–1439), also including a 21 amino acid signal sequence with two primers (rOVGP1F, rOVGP1R), each containing an *Kpn*I or an *Xho*I site. All reverse primers contained a sequence encoding a 6 histidine-tag (Supplementary Material Table S7)^41^.

PCR products were cloned into pcDNA3.1 (+) (Invitrogen, Carlsbad, CA), previously digested with *Eco R*I and *Mun*I (pOVGP1) or *Kpn*I and *Xho*I (rOVGP1). PCR products corresponding to the two truncated C-terminal regions of porcine OVGP1 were cloned into pcDNA3 (+) (Invitrogen, Carlsbad, CA), previously digested with *Hind*III and *Xho*I. pcDNA3.1 vector containing porcine OVGP1 was digested with *Kpn*I and *Hind*III to obtain the first region of the porcine OVGP1 protein (nucleotides 11–757). That protein region was subcloned into pcDNA3 where the two truncated C-terminal regions of porcine OVGP1 had been previously cloned.

The D region (mMBP-pOVGP1d; nucleotides 1454–1594) and DB region (mMBP-pOVGP1bd; nucleotides 1427–1594) of pOVGP1 were PCR amplified using oligonucleotides containing *Not*I and *Xho*I restriction sites (mMBP-pOVGP1df, mMBP-pOVGP1dr, mMBP-pOVGP1bdf, mMBP-pOVGP1bdr). The resulting PCR products, isolated after digestion with *Not*I *and Xho*I were subcloned in frame with the mMBP gene of pHLmMBP-1^15^, a mammalian expression vector derived frompHLsec^42^. Restrictions enzymes were purchased from Fermentas (San Leon-Rot, Germany).

Junction fragments and PCR derived plasmid inserts were verified by DNA sequencing (Molecular Biology Section, Service of Support to the Experimental Sciences (SACE), University of Murcia). Expression plasmids were purified with Gen Eluted Plasmid Kit (Sigma-Aldrich, St. Louis, MO).

### Protein expression and purification

HEK 293T cells were grown in Corning^®^ Roller Bottles (Sigma-Aldrich, St. Louis, MO) (37 °C, 5% CO_2_ and 95% humidity) for 48–72 hr to 80–90% confluence using DMEM medium (Dulbecco’s Eagle Medium, Thermo Fisher Scientific, Rockford, IL) supplemented with 10% fetal bovine serum (Gibco BRL-Life Technologies, Gaithersburg, MD) and 4 mM glutamine (Gibco BRL-Life Technologies, Gaithersburg, MD). To express rOVGP1, pOVGP1 and pOVGP1ab, cells were transfected with polyethylenimine (PEI, Sigma-Aldrich, St. Louis, MO). Conditioned media were harvested 72 hr after transfection, adjusted with 10 mM imidazole, 20 mMNa-HEPES, pH 7.8,150 mMNaCl and incubated with Ni-NTA Superflow (Qiagen, Hilden, Germany) overnight at 4 °C. After washing, proteins were eluted form the nickel beads with 500 mM imidazole, 20 mMNa-HEPES pH 7.8, 150 mMNaCl and subsequently dialyzed against 150 mM TrisHCl, 200 mM NaCl, 10% glycerol. Protein concentration was measured absorbance at 280 nm.

pOVGP1a, mMBP-pOVGP1d and mMBP-pOVGP1bd, as well as unfused control mMBP, were expressed in CHO cells. Cells were grown (37 °C, 5% CO_2_ and 95% humidity) for 24 hr to 70–80% confluence in F-12 medium (Biowest, Nuaillé, France) supplemented with 10% fetal bovine serum and 100 U/ml penicillin-streptomycin (GibcoBRL-Life Technologies, Gaithersburg, MD). Transient transfections were performed with X-tremeGene HP (Roche Applied Science, Indianapolis, IN) in accordance with the manufacturer’s protocol. Conditioned media were harvested at 48 hr, concentrated 100x and adjusted with a 150 mM TrisHCl, 200 mM NaCl, 10% glycerol.

Mass spectrometry of purified recombinant proteins (pOVGP1, pOVGP1ab and rOVGP1) was carried out using a HPLC/MS system, in which an Agilent 1100 Series HPLC (Agilent Technologies, Santa Clara, CA, USA) is connected to an Agilent Ion Trap XCT Plus Mass Spectrometer (Agilent Technologies, Santa Clara, CA, USA) using an electrospray (ESI). The pOVGP1a, mMBP-pOVGP1bd or mMBP-pOVGP1d in the conditional medium of transfected cells was separated by SDS-PAGE and stained with Coomassie. The bands corresponding to each protein were cut out and processed for proteomic analysis. Data processing was performed with Data Analysis program for LC/MSD Trap Version 3.3 (Bruker Daltonik, GmbH, Germany) and Spectrum Mill MS Proteomics Workbench (Rev A.03.02.060B, Agilent Technologies, Santa Clara, CA, USA) by the Molecular Biology Section, Service of Support to the Experimental Sciences (SACE), University of Murcia. Further analysis were performed using the Protein 230 kit for on-chip electrophoresis (2100 Bioanalyzer; Agilent Technologies) where pOVGP1 and rOVGP1 were obtained with a purity of over 90% and 95% respectively.

### Immunoblots

Purified proteins were separated by SDS-PAGE, transferred to PVDF membranes which were probed with Penta-His mouse monoclonal antibody (Qiagen, Hilden, Germany) or a rabbit anti-OVGP1 polyclonal antibody (Abcam, Cambridge, Great Britain) prior to visualization by chemiluminescence. Proteins were treated with N-glycosidase F (Roche^®^, Mannheim, Germany) before separation on SDS-PAGE. Proteins were also stained with Simply Blue^TM^ Safe Stain (Invitrogen, Carlsbad, CA) after SDS-PAGE.

IVM porcine oocytes were incubated for 1 hr at 37 °C in 125 μg/mL of pOVGP1, pOVGP1ab and rOVGP1, lysed and analyzed by immunoblot with a rabbit anti-OVGP1 polyclonal antibody. β-actin was used as a loading control. Average data from three experiments were quantified by image analysis.

### Collection and *in vitro* maturation (IVM) of porcine oocytes

Unless otherwise indicated, all chemicals and reagents were purchased from Sigma-Aldrich Química, Madrid, Spain. Porcine oocytes were isolated from ovaries obtained from 6 to 7 month-old animals slaughtered at an abattoir. The ovaries were transported to the laboratory in saline solution containing 100 μg/mL kanamycin sulfate at 38.5 °C and washed once in 0.04% cetrimide solution (w/v) and twice in saline within 30 minutes of slaughter.

Cumulus-oocyte complexes (COCs) were aspirated from follicles 3–6 mm in diameter using a needle attached to a syringe. The COCs selection was performed with a stereoscopic microscope and based on a homogeneous cytoplasm and a dense and compact cumulus oophorus. Selected COCs were washed twice with PBS supplemented with 1 mg/ml polyvinyl alcohol (PVA) and 0.005 mg/ml red phenol and twice in maturation medium (North Carolina State University solution-37, NCSU-37) previously equilibrated for 3 hr at 38.5 °C under 5% CO_2_ in air and 95% humidity. Groups of 50 COCs were cultured in 500 μl NCSU-37 medium at 38.5 °C supplemented with dibutyryl cAMP, human chorionic gonadotropin (hCG) and pregnant mare’s serum gonadotropin (PMSG). 20–22 hr later, COCs were washed twice in fresh NCSU-37 medium (without dibutyryl cAMP, hCG and PMSG) and were cultured for an additional 20–2 hr.

### Confocal Microscopy

To detect the binding of the different OVGP1 to the ZP of oocytes, histidine tagged pOVGP1, pOVGP1ab and rOVGP1 were added to *in vitro* maturated (IVM) unfixed porcine oocytes at 125 μg/mL diluted in PBS. IVM oocytes were also incubated (1 hr, 37 °C) with medium of transfected cells containing pOVGP1a, mMBP-pOVG1Pbd and mMBP-pOVG1PD. Alternatively, IVM porcine oocytes were incubated for 15 min, 30 min and 1 hr at 37 °C in medium containing pOVGP1 to investigate time-dependent endocytosis.

Oocytes were washed and fixed with 2% paraformaldehyde (Electron Microscopy Sciences, Hatfield, PA). Fixed oocytes were stained with Penta-His mouse monoclonal antibody diluted 1:100 in PBS. Stained oocytes were placed on a chambered slide (20 μl cavity) with Gene Frame (Advanced Biotechnologies, Leatherhead, UK). Oocytes incubated to detect endocytosis were flattened with the coverslip to improve visualization inside the oocyte using confocal microscopy. Samples were analysed with a DM IRE2 confocal microscope (True Confocal Scanner TCS-SP2, Leica Microsystems, Barcelona, Spain). Image analysis was performed using Leica QWin Image analysis software (Leica Microsystems, Barcelona, Spain).

### Electron Microscopy

Oocytes incubated with pOVGP1 and pOVGP1ab were fixed in 1% glutaraldehyde (Serva, Heidelberg, Germany) buffered in sodium cacodylate, pH 7.4, for 2 hr at 4 °C. After extensive washing, the cells were embedded in melted (37 °C) agarose. Samples were dehydrated through a graded series of ethanol and processed for embedding in LR-White resin (L.R. Embedding Media, Polysciences Europe GmbH, Eppelheim, Germany). Ultrathin sections were incubated with an anti-OVGP1 polyclonal antibody from rabbit and protein A-colloidal gold conjugate (1:70 v/v; 15 nm, Utrecht University, Utrecht, The Netherlands) was used to detect signal. Ultrathin sections were counterstained with uranyl acetate followed by lead citrate and imaged with a Jeol JEM-1011 Transmission Electron Microscope (Jeol, Tokyo, Japan).

### Assessment of ZP solubility

The IVM oocytes from sow were incubated 1 hr (37 °C 5% CO_2_) in 125 and 250 μg/mL of pOVGP1 and pOVGP1ab solution and 10, 50 and 125 μg/mL of rOVGP1 diluted in NCSU-37 medium. Then, oocytes were transferred into PBS and placed in 50 μL of 0.5% (wt/vol) pronase solution in PBS. ZPs were continuously observed for dissolution under an inverted microscope equipped with a warming plate at 37 °C. The dissolution time of the zona of each oocyte was recorded as the time between placement of samples in the pronase solution and time at which the zona was no longer visible at 200X magnification. This time is indicated as “ZP digestion time”.

### *In vitro* fertilization (IVF) of matured pig oocytes

After 44 hr of IVM and before insemination, oocytes were mechanically stripped of surrounding cumulus cells by gentle pipetting until they were completely denuded. Cumulus-free oocytes were washed twice in TALP (Tyrode’s albumin lactate pyruvate) medium, transferred in groups of 50 to wells containing 25 μL of pOVGP1 (250 μg/mL), pOVGP1ab (250 μg/mL) or rOVGP1 (50 μg/mL) or TALP (control) and incubated for 60 min at 38.5 °C.

For semen preparation, sperm-rich fractions of semen from boars was collected using gloved-hand method from Artificial Insemination Center (AIC). A volume of 500 μL of this fraction of semen was centrifuged (700 × g, 30 min) through a 45 and 90% (v/v) discontinuous Percoll (GE Healthcare, Buckinghamshire, United Kingdom) gradient. The pellet obtained was resuspended in TALP medium and centrifuged (10 min, 700 × g) and the resultant pellet was resuspended in TALP medium. 475 μL of this suspension was added to wells containing the proteins and oocytes which provided a final volume of 500 μL with 10,000 spermatozoa/mL and pOVGP1 (12.5 μg/mL), pOVGP1ab (12.5 μg/mL), or rOVGP1 (2.5 μg/mL). 3 hr later, putative zygotes were washed in fresh TALP medium and incubated in 500 μL of TALP medium for an additional 15 hr after which they were fixed and stained for assessment of fertility.

### Fixation and staining of putative zygotes and IVF assessment

18 hr post-insemination, putative zygotes were washed in PBS, fixed (30 min) in 0.5% glutaraldehyde (Serva, Heidelberg, Germany), stained (15 min) in 1% Hoechst (Sigma, Madrid, Spain) and mounted on glass slides. Cells were evaluated using a microscope equipped with epi-fluorescence (Leica^®^ DMLS, Barcelona, Spain) at 400X magnification. Fertilization was assessed by the number of spermatozoa attached to the ZP, penetration of the ZP and monospermy.

### Statistical analysis

Statistical analyses were performed using SPSS v.19 (SPSS Inc. Chicago, IL). All data are expressed as the mean ± SEM and were analyzed by one-way ANOVA. When ANOVA revealed a significant effect, values were compared using multiple comparison post-hoc test (Tukey). Differences were considered statistically significant at p < 0.05.

## Additional Information

**How to cite this article**: Algarra, B. *et al*. The C-terminal region of OVGP1 remodels the zona pellucida and modifies fertility parameters. *Sci. Rep*. **6**, 32556; doi: 10.1038/srep32556 (2016).

## Supplementary Material

Supplementary Information
