# Supplementary material for: The C-terminal region of OVGP1 remodels the zona pellucida and modifies fertility parameters
Source: Sci Rep. 2016 Sep 7;6:32556. doi: 10.1038/srep32556 (PMC5013273; doi:10.1038/srep32556)
Supplement: Supplementary Information [file srep32556-s1.pdf]

The C-terminal region of OVGP1 remodels the zona pellucida and modifies fertility parameters

Algarra B [1], Han L [2], Soriano-Úbeda C [3], Avilés M [1], Coy P [3], Jovine L [2], Jiménez-Movilla M [1*]

Affiliations: [1]Department of Cell Biology and Histology, School of Medicine, University of Murcia, Campus Mare Nostrum and IMIB-Arrixaca ,Murcia, Spain.

[2] Department of Biosciences and Nutrition & Center for Innovative Medicine, Karolinska Institutet, Huddinge, Sweden.

[3]Department of Physiology, Faculty of Veterinary, University of Murcia, Campus Mare Nostrum and IMIB-Arrixaca, Murcia, Spain.

*Correspondence to Maria Jimenez-Movilla (mariajm@um.es)

Supplementary Material Fig. S1. Conservation of the C-terminus of OVGP1 among mammals. Alignment of the C-terminus of OVGP1 of fourteen mammalian species labeled with regions A, B, C, D and E. The A region corresponds mainly to the glycosidase hydrolase family 18 domain. The C region is an insert present only in mice and the E region is typical of the human, chimpanzee and orangutan. Modified from Aviles et al., 2010.

Supplementary Material Fig. S2. Recombinant OVGP1 proteins expressed in CHO cells. (a) Porcine recombinant OVGP1 glycoprotein were expressed in mammalian cells (HEK and CHO cells), separated by SDS-PAGE and analyzed by immunoblot using anti-His monoclonal antibodies. **(b)** IVM porcine oocytes were incubated for 60 min, at 37 ºC with medium of transfected CHO cells containing pOVGP1, pOVGP1AB or rOVGP1. Oocytes were fixed and imaged by confocal (upper) and DIC (lower) microscopy using anti-His monoclonal antibodies.

Supplementary Material Fig. S3. Identification of recombinant OVGP1 proteins. Complete sequence of OVGP1 with tryptic peptides identified by MS/MS shown in grey. Purified recombinant proteins (pOVGP1, pOVGP1AB and rOVGP1) were analyzed in a HPLC/MS system consisting of an Agilent 1100 Series HPLC (Agilent Technologies, Santa Clara, CA, USA) connected to an Agilent Ion Trap XCT Plus Mass Spectrometer (Agilent Technologies, Santa Clara, CA, USA) using an electrospray (ESI). Medium of transfected CHO cells containing pOVGP1A, mMBP-pOVGP1BD or mMBP-pOVGP1D

were separated by SDS-PAGE and analyzed by Coomassie. Bands corresponding to each proteins were cut and processed for proteomic analysis like before.

Supplementary Material Fig. S4. Image analysis of the porous area of oocytes incubated with pOVGP1 and rOVGP1. The enclosed area was defined as porous according to the presence of dark pores in the ZP surface. Image analysis was performed using Leica QWin Image analysis software (Leica Microsystems, Barcelona, Spain).

Supplementary Material Fig. S5. Higher magnification electron microscopy. (a) Immunogold transmission electron microscopy (EM) using anti-OVGP1 antibody to detect specific labelled multivesicular-like structures (white asterisk). pOVGP1 was also detected throughout ZP but not in cortical granules (arrows). **(b)** Ultrathin-sections were stained with PNA lectin to detect cortical granules (arrows)**.** Scale bars, 0.25 µm.

Supplementary Material Fig. S6. **Recombinant OVGP1 fragments are not endocytosed in IVM porcine oocytes.** IVM porcine oocytes incubated with pOVGP1, pOVGP1A, mMBP-pOVGP1BD or mMBP-pOVGP1D were fixed and permeabilized. Immunofluorescence (IF) confocal analysis using anti6His monoclonal antibodies.

Supplementary Material Table S7. DNA primer sequences.

Supplementary material Fig. S1

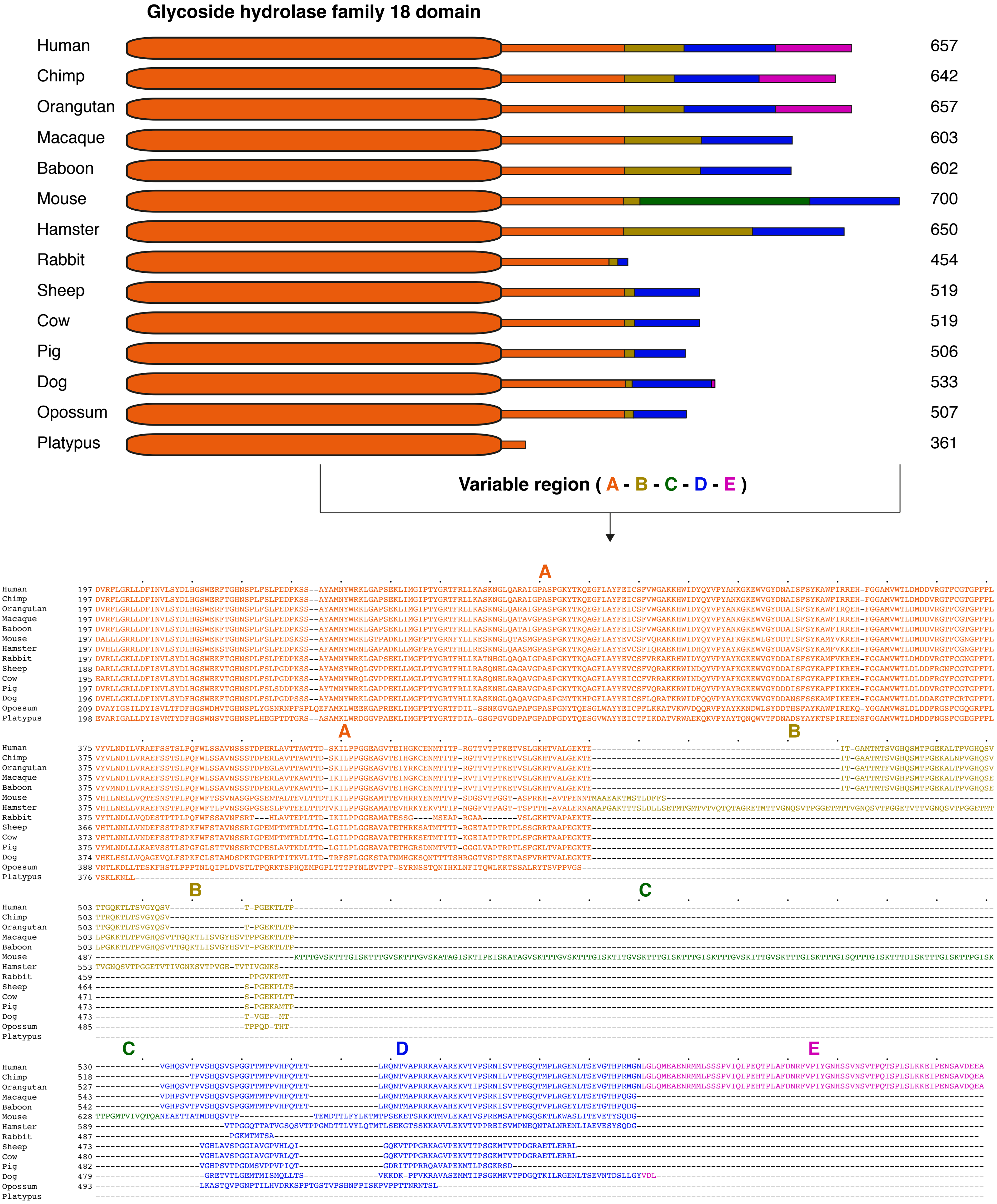

Supplementary material Fig. S2

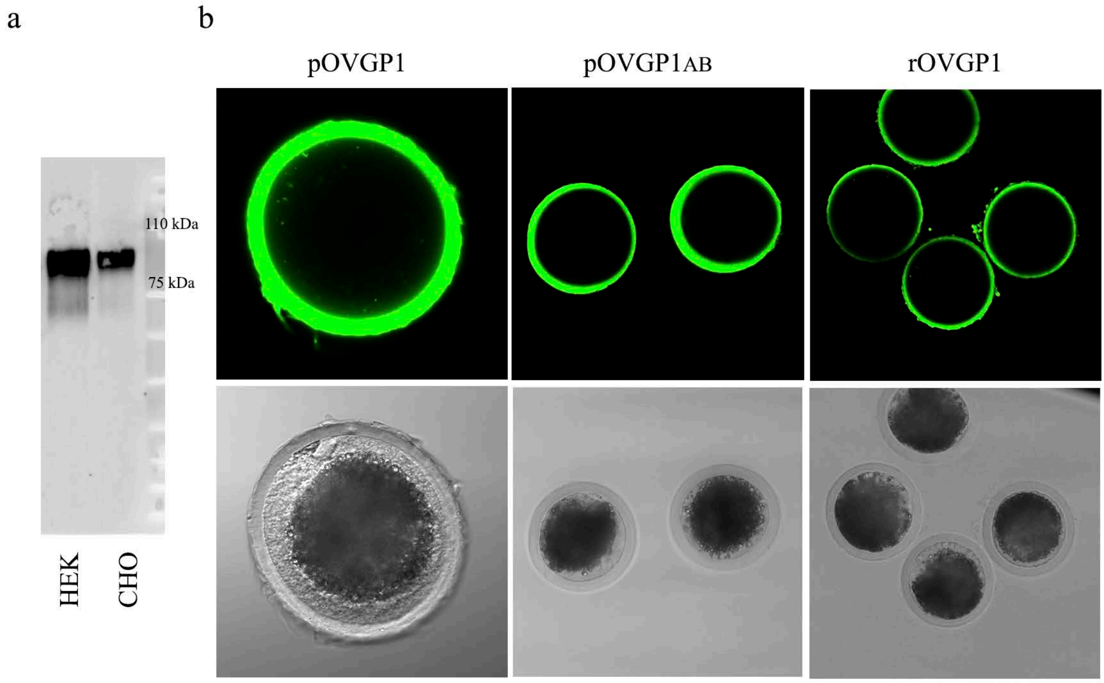

Supplementary material Fig. S3

pOVGP1

MGKLLLWVGLVLVLKHHNGAAHKLVCYFANWAFSRPGPASILPRDLDPFLCTHLVFAFAS
MNDSQIVAKDARDESIFYPEFNQLKERNEKLKTLLSIGGWNFGTSRFTTMLSTFTNREKF
IRSAIGLLRTHGFDGLDLFFLYPGLRGSPRRDRWNFLFLLEELLLAFRREAQLTMRPRLL
LSAAVSADPHVIQKAYDVRLLGRLLDFINVLSYDLHGSWEKVTGHNSPLFSLSDDPKSSA
YTMNYWRKLGAPPEKLLMGFPTYGRTFRLLKASKNELGAEAVGPASPGKYTKQAGFLAYY
EVCSFVQRAKKRWIDHQYVPYAYRGKEWVGYDDDISFSYKAFFIKKEHFGGAMVWTLDLD
DVRGTFCGTGPFPLVYMLNDLLLKAEVSSTLSPGFGLSTTVNSSRTCPESLAVTKDLTTD
LGILPLGGEAVATETHGRSDNMTVTPGGGLVAPTRPTLSFGKLTVAPEGKTESPGEKAMT
PVGHPSVTPGDMSVPPVPIQTGDRITPPRRQAVAPEKMTLPSGKRSD

pOVGP1AB

MGKLLLWVGLVLVLKHHNGAAHKLVCYFANWAFSRPGPASILPRDLDPFLCTHLVFAFAS
MNDSQIVAKDARDESIFYPEFNQLKERNEKLKTLLSIGGWNFGTSRFTTMLSTFTNREKF
IRSAIGLLRTHGFDGLDLFFLYPGLRGSPRRDRWNFLFLLEELLLAFRREAQLTMRPRLL
LSAAVSADPHVIQKAYDVRLLGRLLDFINVLSYDLHGSWEKVTGHNSPLFSLSDDPKSSA
YTMNYWRKLGAPPEKLLMGFPTYGRTFRLLKASKNELGAEAVGPASPGKYTKQAGFLAYY
EVCSFVQRAKKRWIDHQYVPYAYRGKEWVGYDDDISFSYKAFFIKKEHFGGAMVWTLDLD
DVRGTFCGTGPFPLVYMLNDLLLKAEVSSTLSPGFGLSTTVNSSRTCPESLAVTKDLTTD
LGILPLGGEAVATETHGRSDNMTVTPGGGLVAPTRPTLSFGKLTVAPEGKTESPGEKAMT
P

rOVGP1

MGRLLLWLGLVLVLDCHDGAAYKLVCYFTNWAHSRPGPAAILPHDLDPFLCTHLIFAFAS
MNDNEIVAKDVQDERIFYPEFNKLKERNRELKTLLSIGGWNFGTTRFTAMLSSFASREKF
INSVISLLRTHNFDGLDLFFLYPGLRGSPAHDRWTFLFLVEELLFAFQREALLIKRPRLL
LSAAVSGVPHIIQTSYDVRLLGKLLDFINVLSYDLHGSWEKFTGHNSPLFSLPEDPKSSA
YAMNYWRKLGAPSEKLIMGFPTYGRTFHLLKATNHGLQAQAIGPASPGKYTKQAGFLAYY
EVCSFVRKAKRHWIDYQYVPYAYKGKEWVGYDDAISFSYKAMFVKREHFGGAMVWTLDMD
DVRGTFCGNGPFPLVYTLNDLLVQDESTPTPLPQFWFSSAVNFSRTHLAVTEPLTTDIKI
LPPGGEAMATESSGMSEAPRGAAVSLGKHTVAPAEKTEPPGVKPMTPGKMTMTSA

pOVGP1A

MGKLLLWVGLVLVLKHHNGAAHKLVCYFANWAFSRPGPASILPRDLDPFLCTHLVFAFAS
MNDSQIVAKDARDESIFYPEFNQLKERNEKLKTLLSIGGWNFGTSRFTTMLSTFTNREKF
IRSAIGLLRTHGFDGLDLFFLYPGLRGSPRRDRWNFLFLLEELLLAFRREAQLTMRPRLL
LSAAVSADPHVIQKAYDVRLLGRLLDFINVLSYDLHGSWEKVTGHNSPLFSLSDDPKSSA
YTMNYWRKLGAPPEKLLMGFPTYGRTFRLLKASKNELGAEAVGPASPGKYTKQAGFLAYY
EVCSFVQRAKKRWIDHQYVPYAYRGKEWVGYDDDISFSYKAFFIKKEHFGGAMVWTLDLD
DVRGTFCGTGPFPLVYMLNDLLLKAEVSSTLSPGFGLSTTVNSSRTCPESLAVTKDLTTD
LGILPLGGEAVATETHGRSDNMTVTPGGGLVAPTRPTLSFGKLTVAPEGKTE

mMBP-pOVGP1BD

mMBP-SPGEKAMTPVGHPSVTPGDMSVPPVPIQTGDRITPPRRQAVAPEKMTLPSGKRSD

mMBP-pOVGP1D

mMBP-VGHPSVTPGDMSVPPVPIQTGDRITPPRRQAVAPEKMTLPSGKRSD

Supplementary material Fig. S4

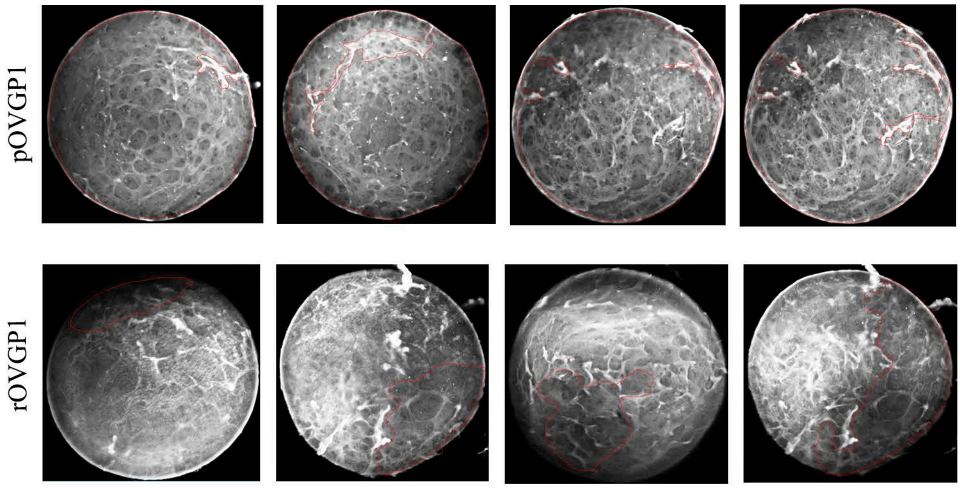

Supplementary material Fig. S5

a

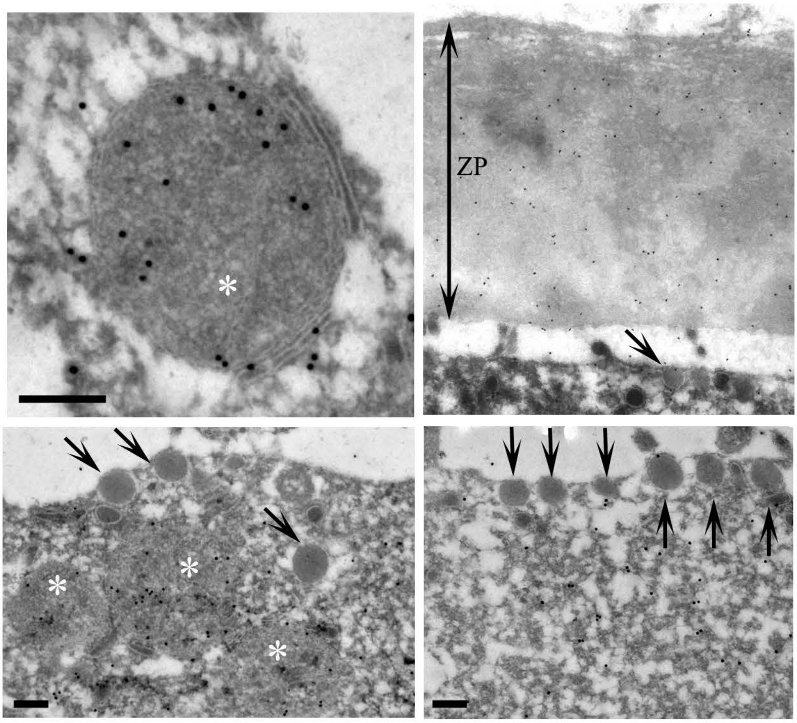

b

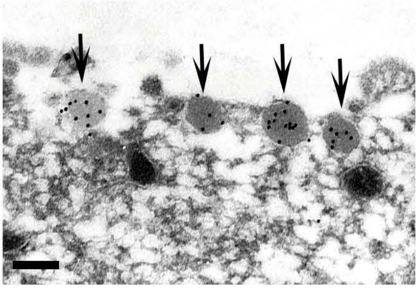

Supplementary material Fig. S6

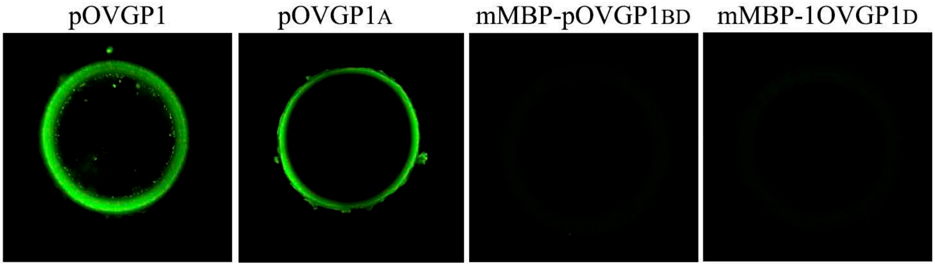

Table S7. Primers design for cloning.

**Primer name**	**Sequence 5’→3’**
**pOVGP1**	
pOVGP1F	gatcaggtaccatggggaagctgttgctgtgggtcgg
pOVGP1R	aagcacaattgttaatgatgatgatgatgatggtctgacctcttcccagaggggagagtcatc
**pOVGP1AB**	
pOVGP1ABF	gggcccaagcttggggcgcccccg
pOVGP1ABR	gcgctcgagtcaatgatgatgatgatgatgaggggtcatggccttctctccag
**pOVGP1A**	
pOVGP1AF	gggcccaagcttggggcgcccccg
pOVGP1AR	gggcccctcgagtcaatgatgatgatgatgatgctcagtcttcccttctggagcgaca
**mMBP-pOVGP1D**	
mMBP-pOVGP1DF	aaggaaaaaagcggccgcggtgggccatccgtctgtg
mMBP-pOVGP1DR	ccgctcgaggtctgacctcttcccaga
**mMBP-pOVGP1BD**	
mMBP-pOVGP1BDF	aaggaaaaaagcggccgcgagccctggagagaaggcc
mMBP-pOVGP1BDR	ccgctcgaggtctgacctcttcccaga
**rOVGP1**	
rOVGP1F	cgcggtaccatggggaggctgttgctg
rOVGP1R	gggcccctcgagtcaatgatgatgatgatgatgagcagaggtcatcgtcatcttg
